# Towards a radiation free numerical modelling framework to predict spring assisted correction of scaphocephaly

**DOI:** 10.1080/10255842.2023.2294262

**Published:** 2023-12-18

**Authors:** Begona Garate Andikoetxea, Sara Ajami, Naiara Rodriguez-Florez, N. U. Owase Jeelani, David Dunaway, Silvia Schievano, Alessandro Borghi

**Affiliations:** a University College London, United Kingdom; b Great Ormond Street Hospital, London, United Kingdom; c Universidad de Navarra, Spain; d Ikerbasque, Spain; e Department of Engineering, Durham University, Durham, United Kingdom

**Keywords:** Finite element modelling, craniosynostosis, spring cranioplasty

## Abstract

Sagittal Craniosynostosis (SC) is a congenital craniofacial malformation, involving premature sagittal suture ossification; spring-assisted cranioplasty (SAC) – insertion of metallic distractors for skull reshaping – is an established method for treating SC. Surgical outcomes are predictable using numerical modelling, however published methods rely on computed tomography (CT) scans availability, which are not routinely performed. We investigated a simplified method, based on radiation-free 3D stereophotogrammetry scans. Eight SAC patients (age 5.1 ± 0.4 months) with preoperative CT and 3D stereophotogrammetry scans were included. Information on osteotomies, spring model and post-operative spring opening were recorded. For each patient, two preoperative models (PREOP) were created: i) CT model and ii) S model, created by processing patient specific 3D surface scans using population averaged skin and skull thickness and suture locations. Each model was imported into ANSYS Mechanical (Analysis System Inc., Canonsburg, PA) to simulate spring expansion. Spring expansion and cranial index (CI - skull width over length) at times equivalent to immediate postop (POSTOP) and follow up (FU) were extracted and compared with in-vivo measurements. Overall expansion patterns were very similar for the 2 models at both POSTOP and FU. Both models had comparable outcomes when predicting spring expansion. Spring induced CI increase was similar, with a difference of 1.2%±0.8% for POSTOP and 1.6%±0.6% for FU. This work shows that a simplified model created from the head surface shape yields acceptable results in terms of spring expansion prediction. Further modelling refinements will allow the use of this predictive tool during preoperative planning.

## UNSTRUCTURED ABSTRACT

Spring-assisted cranioplasty (SAC) –insertion of metallic distractors helping skull reshaping – is a method for treating sagittal craniosynostosis, caused by premature sagittal suture closure. We present a method for predicting SAC outcomes, relying on radiation-free 3D stereophotogrammetry scans. Eight patients with preoperative CT and 3D stereophotogrammetry scans were recruited; results of spring expansion simulation were compared between models created using CT versus 3D scan data. Expansion patterns and extent of reshaping were very similar. This work proves that SAC preoperative planning can be carried out using non-ionising imaging. Further modelling refinements will allow clinical adoption of this predictive tool.

## 1. Introduction

Craniosynostosis, which affects one in every two thousand live births (Fearon 2014), is a medical condition where cranial sutures (synarthroses connecting the bones of the head with each other through a fibrous sutural ligament (Savoldi et al. 2018)) ossify prematurely, causing the new-borns’ skull to develop abnormally. Skull sutures help skull moulding during birth and have an important role in shaping cranial shape during the first few years after birth.

Scaphocephaly is the most common type of craniosynostosis, characterised by premature fusion of sagittal suture. This inhibits the skull growth perpendicularly to the affected suture and leads to a narrow and elongated skull shape. If left untreated, this cranial deformity can cause learning and development difficulties: (Kapp-Simon et al. 2007; Knight et al. 2014) works in the literature have reported that babies affected by single suture craniosynostosis (SSC) have a rate of neurocognitive risk between 35% and 50% (Kapp-Simon et al. 2007; Knight et al. 2014) and the prevalent hypothesis is that cranial vault distortion and brain mass restriction cause localised anatomical abnormalities which are not always normalised following surgery. A second factor which is believed to be connected to neurological impairment is elevated intra-cranial pressure (ICP), which although has been reported in up to 20% of the cases of SSC (Bristol et al. 2011), has not yet been proved to be associated with neurodevelopment. Other complications include breathing (such as apnoea), sleeping and eating disorders (Wilkie et al. 2010; Zakhary et al. 2014)

Spring-assisted cranioplasty (SAC) - performed in Great Ormond Street Hospital (GOSH) since 2008 (Rodgers et al. 2017) - has become a well-recognized option for treating scaphocephaly in new-borns. Two bony cuts (parasagittal osteotomies) are performed parallel to the fused suture and two metallic distractors (springs) are pre-crimped and inserted in the calvarium to expand and reshape the patient’s calvarium. Three spring models are available (S10, S12, S14), having the same dimensions but with increasing wire thickness for higher stiffness (1.0-, 1.2- and 1.4-mm wire diameter) (Borghi et al. 2017). Springs are removed between 3 and 6 months after insertion (Rodgers et al. 2017) but are known to achieve full expansion within the first 10 days (Borghi et al. 2017). SAC is novel technique which has only recently been accepted as a suitable alternative to more invasive reshaping techniques (Sharma et al. 2018). Due to the less invasive nature of this technique, the short operative time and the minimal need for transfusion (Rodgers et al. 2017), several craniofacial centres worldwide have adopted it as preferential method to treat babies presenting before 9 months of age, while others have not yet adopted it due to the lack of control on the distraction post-insertion, in terms of final distraction (extent of bony fragment separation) as well as distraction vector and distraction forces in comparison with standard linear cranial distractors (Nowinski et al. 2012). Our group showed that surgical outcomes can be predicted using finite element analysis (FEA) (Borghi et al. 2018; Borghi et al. 2020; Jeelani et al. 2020; Deliège et al. 2021). However, the current method relies on the availability of computed tomography (CT) scans, which is not routinely performed in our and other craniofacial centres (Rodgers et al. 2017, Abdel-Alim et al. 2021) and are usually minimized on patients below the age of 2 years (Pearce et al. 2012) due to the harmful effect of radiations in the paediatric population. On the other hand, the use of 3D surface scans, which allow non-invasive, non-ionising surface imaging, has become common for the preoperative and postoperative assessment of craniofacial patients (Tenhagen et al. 2016; Rodriguez-Florez et al. 2017; Rodriguez-Florez et al. 2020; Abdel-Alim et al. 2021)

In this work, our group investigated a method for creating a simplified model for predicting the SAC outcomes, which only relies on the availability of head surface details, retrievable from radiation-free 3D stereophotogrammetry scans.

## 2. Methodology

### 2.1. Surgical procedure

Following parental consent, 8 patients were included for this study (R&D n.14DS25, REC 15/LO/0386 – NHS Health Research Authority, Bristol BS1 2NT). Each patient received two springs (one positioned anteriorly, one posteriorly). The devices were manufactured by The Active Spring Company UK (TASCUK, Thaxted, UK) using stainless steel wire; three models are available (S10, S12, S14) with increasing wire thickness and consequently increasing stiffness. Each spring has a nominal maximum size equal to 60 mm. Details on the surgical procedure are provided in previous publications by our group (Tenhagen et al. 2016; Rodgers et al. 2017) and details on the mechanical properties of these springs are provided in Borghi et al. (2018). Individual patients’ spring model is provided in Table 1.

**Table 1.** Summary of the patient population used in this study and spring distractors implanted during surgery.

PATIENT	AGE AT CT [months]	AGE AT SURGERY [months]	ANTERIOR SPRING MODEL	POSTERIOR SPRING MODEL
P1	4.3	5.5	S12	S10
P2	3.9	5.0	S14	S14
P3	1.8	4.3	S14	S12
P4	3.0	4.8	S12	S12
P5	3.2	5.6	S12	S12
P6	3.7	5.5	S12	S12
P7	1.4	5.3	S12	S12
P8	1.7	5.2	S12	S12

### 2.2. Image acquisition

Each patient presented with a preoperative CT scan as well as a 3D scan acquired in theatre immediately before spring insertion using a 3D handheld surface scanner (M4D Scanner, Rodin4D, Pessac, France). Details on the 3D scan acquisition and processing can be found in Tenhagen et al. (2016). CT scans were acquired in supine position. Pixel size ranged from 0.2 mm to 0.43 mm; interslice distance ranged from 0.3 and 1.0 mm. For each patient, two preoperative models (PREOP) were created, one by extracting 3D anatomical models from CT - “CT model” - and one by creating a skull model from the 3D stereophotogrammetry scan – “S(urface scan) model”. Information on surgical osteotomies, spring model and location were retrieved in surgery for each patient. Information on on-table spring opening was also retrieved during surgery while spring opening at follow up was extracted from X-ray following a previously described protocol (Borghi et al. 2020).

### 2.3. CT model

A detailed explanation of this method (including the protocol to account for head growth between CT scan and surgical procedure) can be found in Borghi et al. (2018; Borghi et al. 2020).

**Figure 1.**
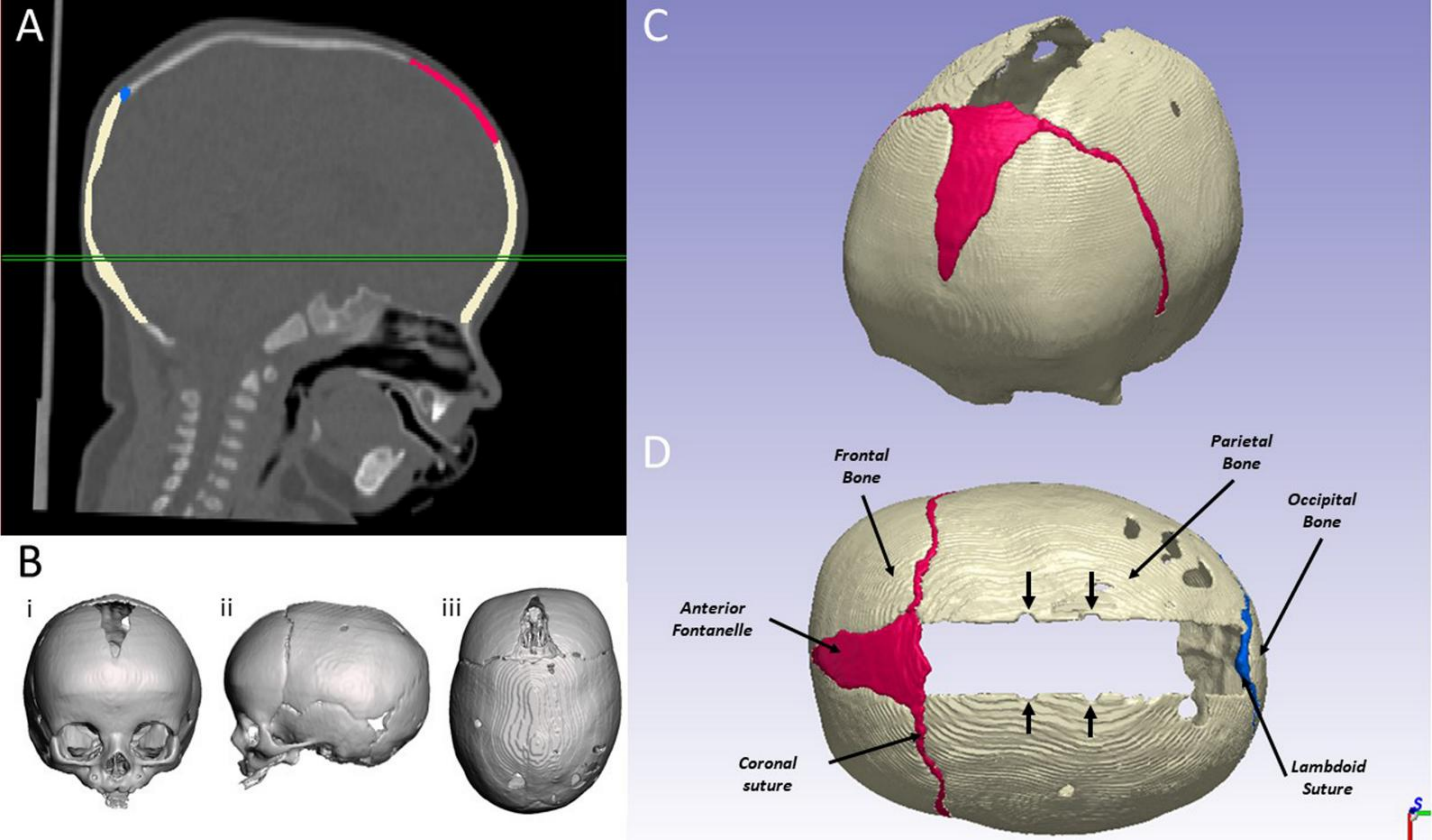
Creation of the CT model from preoperative CT scans: A) image segmentation of a representative patient, with B) front(i), side (ii) and top (iii) view of the patient skull; C) isometric and D) top view of the CT model with osteotomies and spring notches (indicated with arrows).

Briefly, each CT dataset was processed (using ScanIP®, Synopsys, Mountain View, CA, USA – Figure 1A) to create a patient specific geometrical 3D model of the skull, comprising the skull, the anterior fontanelle and the coronal and lambdoid sutures. The model was cut through a plane encompassing the skeletal nasion and the upper border of the left and right auditory meatus (Sadrameli and Mupparapu 2018). Growth between the time of CT and the time of surgery was taken into account by performing linear rescaling, following the method reported in Borghi et al. (2020). Bony cuts were replicated in the model using ScanIP® to simulate the surgical procedure (Figure 1B and C) including the notches corresponding to the spring insertion sites (LAT, A, B, Figure 1C and D). Tetrahedral mesh was created and imported into ANSYS Mechanical.

### 2.4. S model

#### 2.4.1. Hard and soft tissue thickness

**Figure 2.**
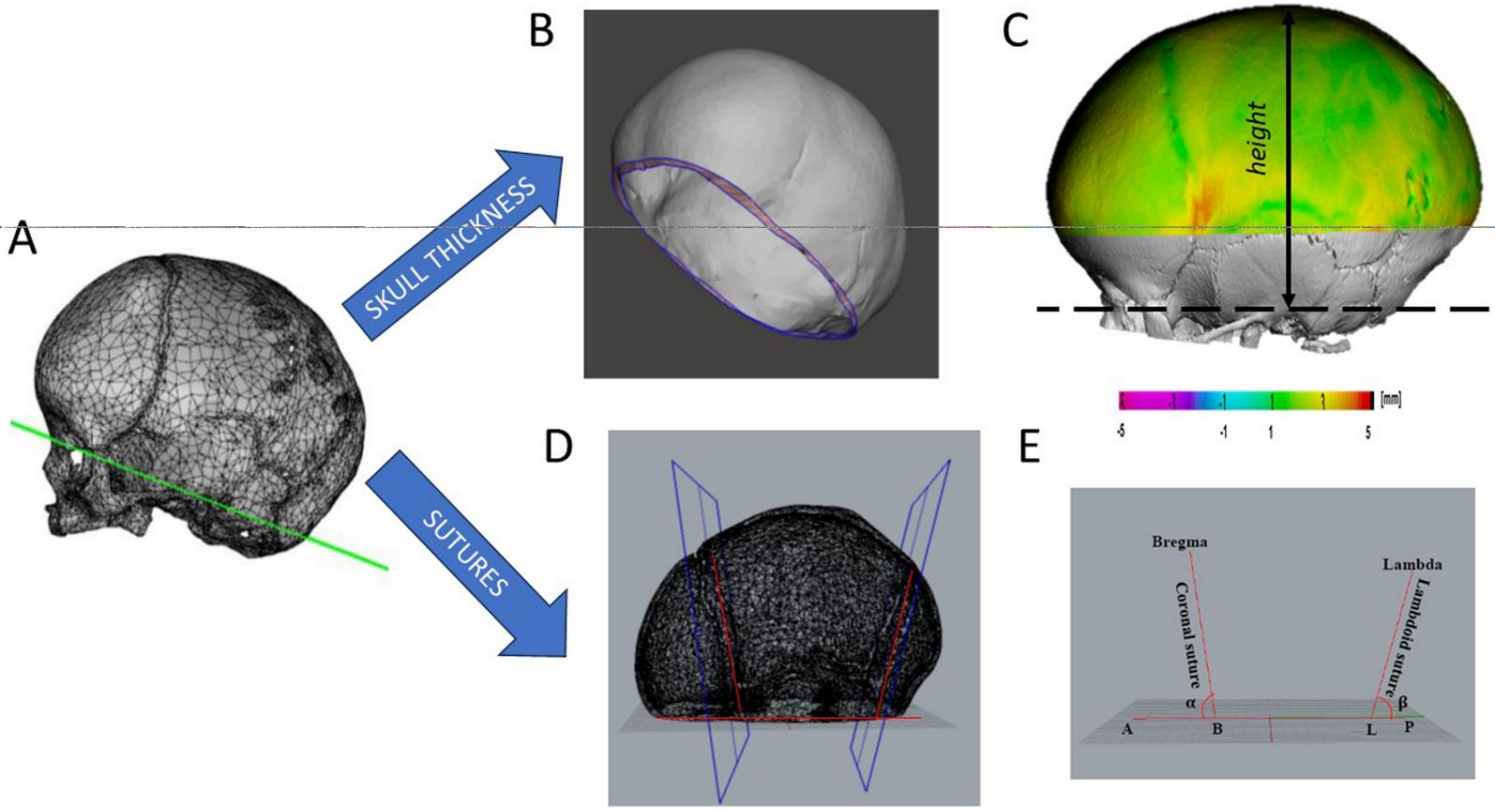
Step for measuring patient specific skin thickness and suture location: A) the patient skull model is cut along a plane passing through the nasion and the top of the left and right auditory meatus; B) the inner and outer surface of the skull are separated; C) skull thickness of the top 75% of the skull height is calculated as surface distance; D) planes running along the coronal and lambdoid sutures are identified; E) measurement of suture plane angles and location.

For each patient STL models of the skull and external soft tissues were extracted using ScanIP’s function “Surface Model” using default settings and applying a triangle reduction of 50% to avoid unnecessary large output model; the skull model was cut along the plane encompassing the nasion and upper border of the left and right auditory meatus (Figure 2A) and processed to extract inner and outer surface of the skull: briefly cranial sutures and skull defects were filled using a combination of commands in Meshmixer (Autodesk, San Francisco, CA) (“fill”,”bridge”) until the inner and outer tab could be fully separated (Figure 2D). The model was further cut along a plane parallel to that passing through that encompassing nasion and left and right auditory meatus and raised by 25% of the overall skull height (measured from the nasion-tragion plane, as in shown in Figure 2C), to ensure that skull base and orbits were not included in the skull thickness measurements (Figure 2C). The skin model was cut along the same plane. Tissue thickness (soft tissue thickness, skull thickness) were calculated as local surface distance (between the inner and outer tab of the skull for the hard tissue thickness between the skin and outer tab of the skull for the soft tissue thickness) and averaged for each patient.

#### 2.4.2. Suture location

To identify the suture location (Figure 2D) for every patient, on each skull model the most anterior point lying on the nasion-tragions plane (point A) and the most posterior point lying on the same plane (point P) were first identified; afterwards, the cranial Bregma and the cranial Lambda (Sadrameli and Mupparapu 2018) were identified. Two planes were created, both perpendicular to the symmetry plane passing through the segment AP, one passing through the Bregma (coronal plane) and one passing through the Lambda (lambdoid plane). Two extra points were identified (Figure 2E), the point B (intersection between the coronal plane and the segment AP) and the point L (intersection between the lambdoid plane and the segment AP). Two angles were defined, the coronal angle α (A-B-Bregma) and the lambdoid angle β (P-L-Lambda). The ratio between AB and AP (AB%) and LP and AP (LP%) were calculated and averaged throughout the patient group (AB%_avg_, AP%_avg_). Similarly, α and β were averaged (α _avg_, β _avg_).

#### 2.4.3. Skull model creation

**Figure 3.**
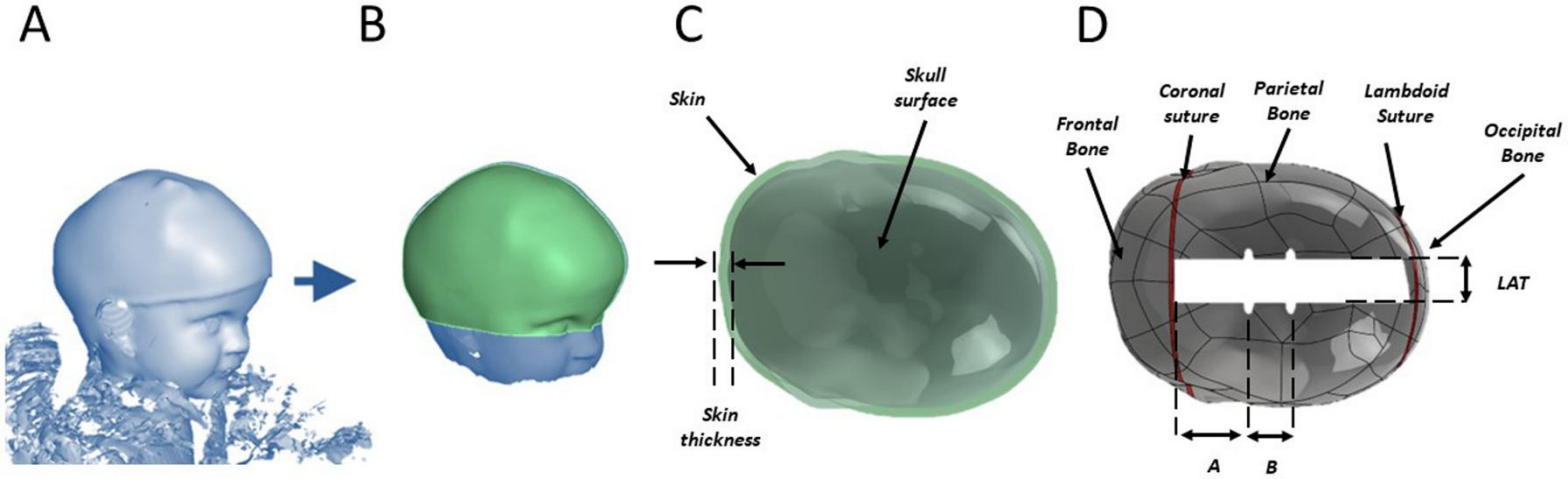
Creation of the S model from preoperative 3D scan: A) 3D scan; B) the head surface is cut along a plan passing through the nasion and the left and right tragions C) the head surface is offset by a constant thickness equal to the population averaged skin thickness value; D) coronal and lambdoid suture are created using population averaged values and osteotomy and spring notches are created.

Preoperative patient 3D surface scans were acquired (Figure 3A) and STL models extracted and processed in Meshmixer®. Each model was cut along a plane passing through the nasion and left and right tragions (Figure 3B). Each model was imported in Solidworks® (Dassault Systems, Vélizy-Villacoublay, France) and 3D nurbs were created. The patient group average thickness (as estimated above) of the soft tissue was used to offset each model to derive an estimated skull surface. For each skull surface, the segment AP (see above) was identified, B and L point created by splitting the AP into segment AB = AB%_avg_ * AP and LP = LP%_avg_ * AP. Two planes were created perpendicular to the skull model midline, one at an angle equal to α_avg_ and one at an angle equal to β_avg_. Such planes were used to split each skull model into five sections: the frontal bone, the coronal suture, the parietal bone, the lambdoid suture, the occipital bone. Coronal suture and lambdoid suture were assumed to be 2 mm wide (Erasmie and Ringertz 1976). Bony cuts and spring notches (LAT, A, B) were replicated in the model.

### 2.5. Simulation of spring expansion

Similarly to Borghi et al. (2018; Borghi et al. 2020), the CT model and S model were imported into ANSYS Mechanical. The S model was imported into ANSYS Mechanical as surface and a homogeneous thickness equal to the average group skull thickness (as estimated above) was assigned for the simulation. Material properties (elastic and viscoelastic properties) for skull and sutures were assigned as in Borghi et al. (2020).

For both models, the lower surface was fully constrained to mimic the tethering of the skull base. Springs were modelled using linear conditions as in Borghi et al. (2018); for each patient anterior and posterior springs parameters were selected according to the spring model used during surgery (Borghi et al. 2018).

### 2.6. Experimental comparison

Spring opening was simulated over 5 days for the CT (OP_CT_) and S (OP_S_) models: the spring opening values were extracted at t = 1s, equivalent to the immediate postoperative spring opening (OP_CT_^POSTOP^_,_ OP_S_^POSTOP^) and at *t* = 5 days, assumed to be equivalent to the expansion at the time of second follow up (OP_CT_^FU^_,_ OP_S_^FU^), and compared with the values measured (M) during surgery (OP_M_^POSTOP^) and from follow-up X-ray (OP_M_^FU^) using the method previously published by our group (Borghi et al. 2017; Borghi et al. 2018; Borghi et al. 2020; Jeelani et al. 2020). All spring opening values (measurements, FEA predictions) are expressed as % of the nominal size of the spring distractors (60 mm). Cranial Index (CI, defined as post-insertion skull biparietal dimension BPD over occipitofrontal dimension OFD – Figure 6) was calculated and compared for the two model for each patient in the undeformed configuration (PREOP) t = 1s (POSTOP) and *t* = 5 days (FU). Correlation between results (in terms of CI) of the CT and S models was assessed and quantified in terms of Spearman correlation coefficient ρ.

## 3. Results

Eight patients were included in this study: each received both a CT (age at CT = 2.9 ± 1.1 months) and a 3D scan at the time of surgery (age at surgery = 5.1 ± 0.4 months). All patients were affected by sagittal craniosynostosis. All patients were male. Each patient was treated for scaphocephaly correction and two springs were implanted. Table 1 shows a summary of the patients details and the distractors used. Following a previously published protocol (Borghi et al. 2017), data relative to spring expansion was recorded at the time of surgery and extracted from follow-up X-rays.

**Figure 4.**
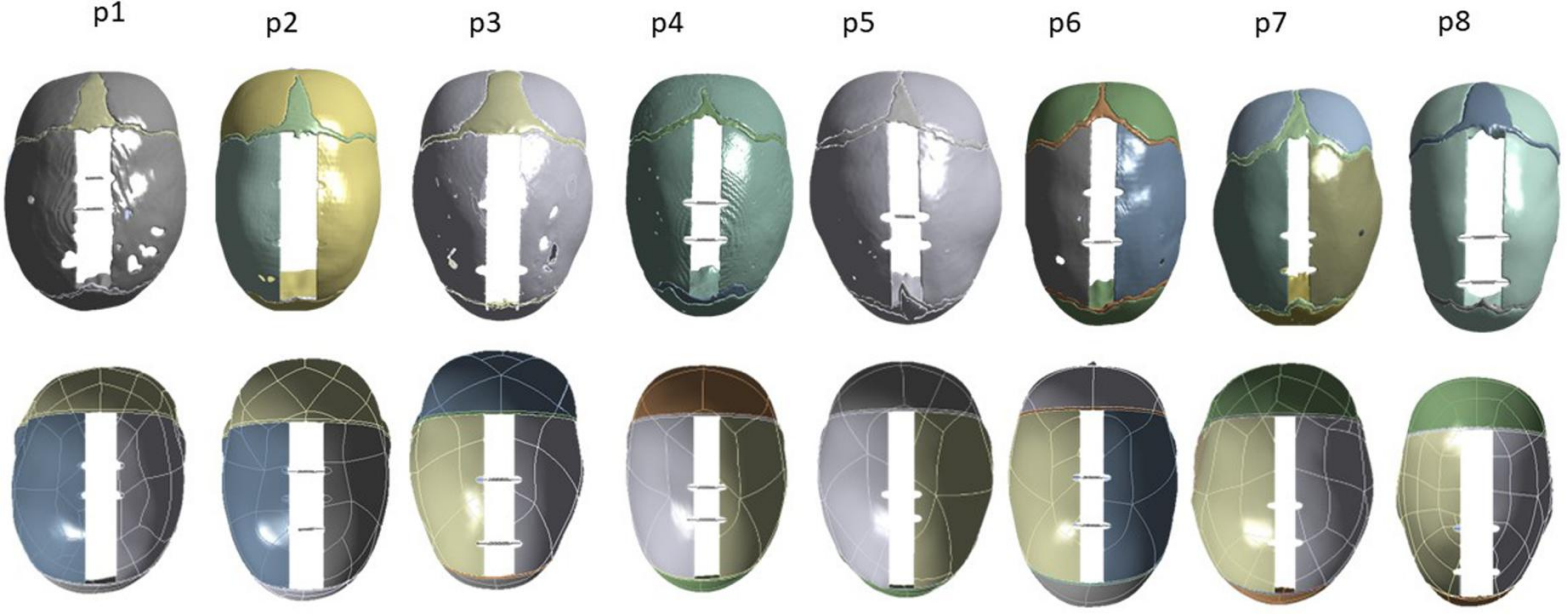
Comparison of the CT model (top row) and S model (bottom row) for each patient.

Average soft tissue thickness in this patient group was 2.8 mm ± 0.4 mm while average skull thickness was 2.5 mm ± 0.4 mm. Average population values of coronal and lambdoid angles were respectively 83 ± 4° and 68 ± 3°. Figure 4 shows a comparison between the CT model and the S model for each patient of the population, showing visual good matching between the two models.

**Figure 5.**
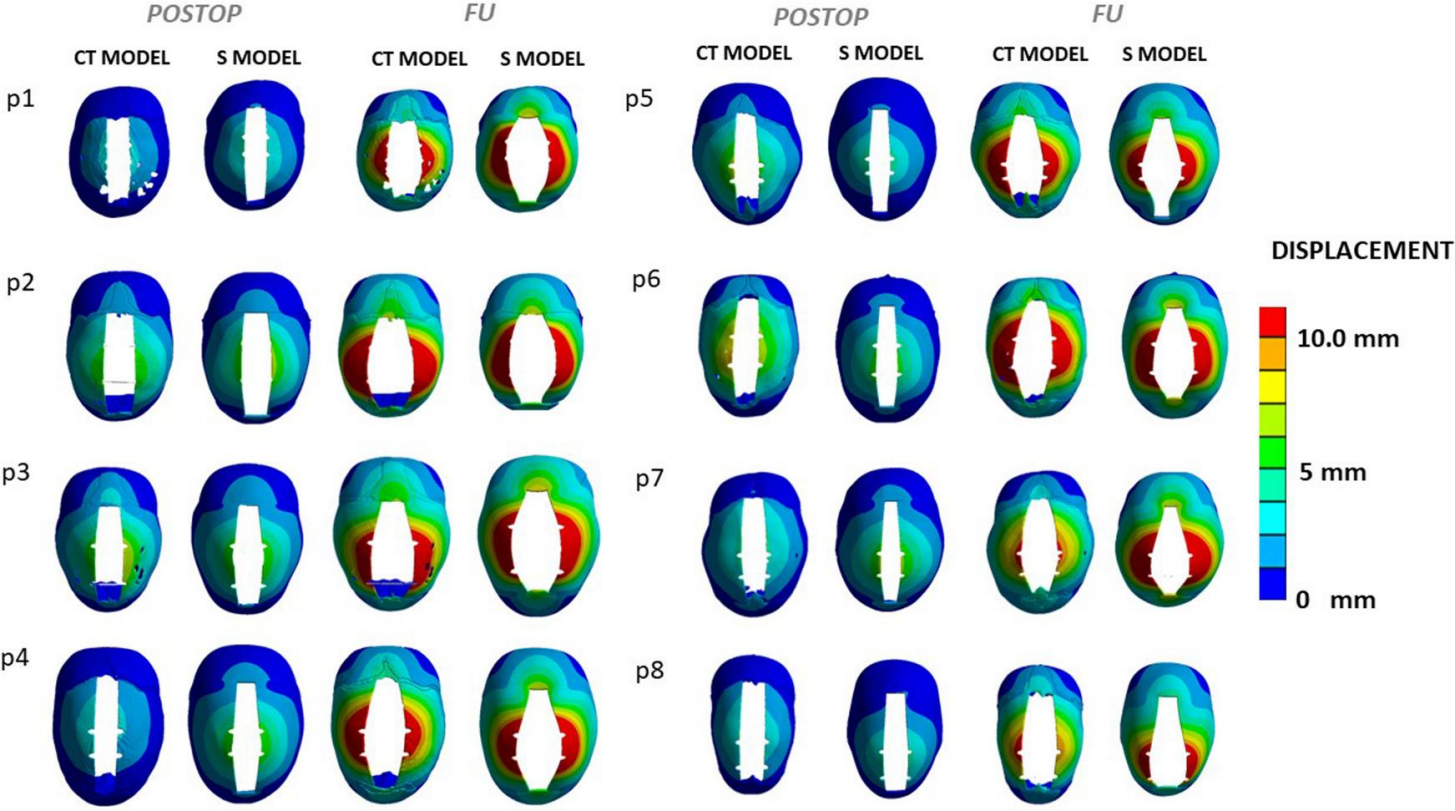
Comparison of the post-expansion skull shape for the CT model and S model for the SAC patient group, at POSTOP and FU.

On-table spring expansion was simulated for each patient (data related to computational meshes is provided in Table 2): Figure 5 shows a comparison of the simulated expansion for three representative patients. Spring expansion was compared between the two groups of simulations and the spring measurements obtained during surgery: the spring expansion was compared at postop and follow up, with an average error of 4.0% ± 1.3% for the CT model and 3.9% ± 1.8% for the S model at POSTOP, and an average error of 5.5% ± 4.2% for the CT model and a 3.6% ± 2.3% at FU.

**Table 2.** Summary of the nodes (with node density as nodes/mm3) and elements for each FE model.

	CT MODEL		S MODEL	
PATIENT	NODES (density)	ELEMENTS	NODES (density)	ELEMENTS
P1	101219 (0.91)	335713	11579 (0.11)	11080
P2	131619 (1.02)	452300	11797 (0.11)	11268
P3	121319 (1.22)	424671	11120 (0.11)	10560
P4	138738 (1.06)	471725	11522 (0.11)	11012
P5	144880 (1.41)	501602	12297 (0.11)	11723
P6	77208 (0.79)	251476	12029 (0.11)	11682
P7	95264 (0.91)	310454	13734 (0.11)	13098
P8	46509 (0.36)	157144	12260 (0.11)	11690

**Figure 6.**
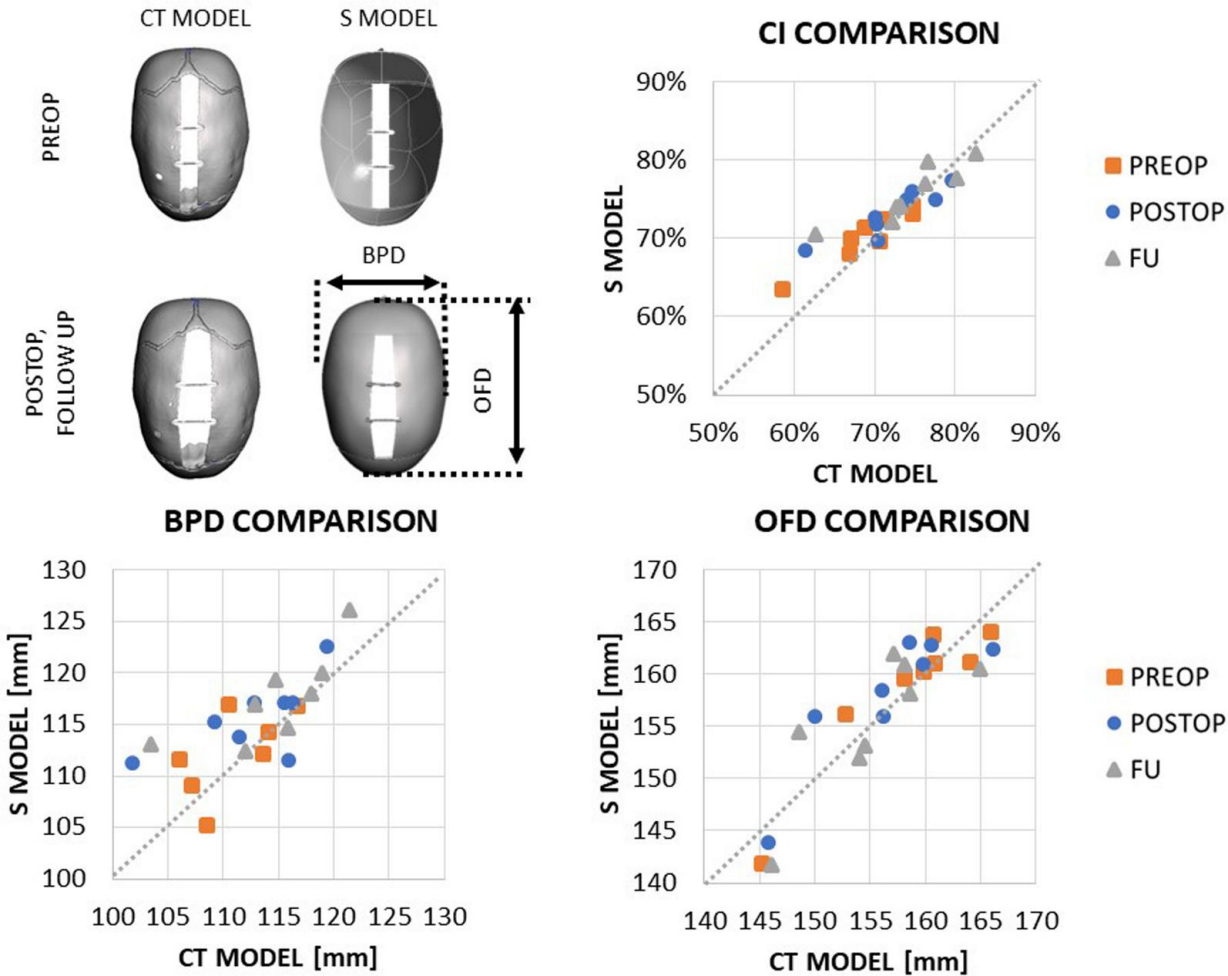
Comparison between model CI at PREOP, POSTOP and FU between the CT model and the S model.

When assessing the preoperative and predicted postoperative BPD, OFD and CI values (Figure 6), the absolute difference between the two model’s CI predictions was respectively 2.0% ± 1.5% at PREOP, 2.4% ± 2.1% at POSTOP and 1.6% ± 0.6% at FU. Good correlation between CI values in the CT and S model was found at PREOP (ρ = 0.93, *p* = 0.002), POSTOP (ρ = 0.98, *p* < 0.001) and FU (ρ = 0.86, *p* = 0.008). The difference in BPD was respectively 2.0 ± 3.9 mm at PREOP, 3.1 mm ± 4.1 mm at POSTOP and 2.9 mm ± 3.5 mm at FU. The difference in OFD was respectively 0 mm ± 2.6 mm at PREOP, 1.4 mm ± 3.2 mm at POSTOP and 0.1 mm ± 4.0 mm at FU.

## 4. Discussion

In this paper, we present a novel method to create patient specific simplified skull models, suitable for the prediction of spring expansion in babies undergoing SAC.

Our group has produced, validated, and optimized a numerical model which allows precise estimation of cranial adaptation post-spring insertion (Borghi et al. 2018; Borghi et al. 2020; Bozkurt et al. 2020; Bozkurt et al. 2020; Deliège et al. 2021). Such modelling technique requires data from tomographic imaging such as CTs or MRI to replicate patient specific anatomy. For patients who undergo SAC, only a small subset of subjects (1 in 4 in GOSH) receive preoperative imaging as the diagnosis is performed with clinical examination (Fearon 2014) and early exposure to ionizing imaging (with an effective dose ranging between 2.3 and 4.3 mSv for a paediatric low dose head CT (Didier et al. 2010; Livingston et al. 2014; Obara et al. 2017)) is generally avoided as it may cause tumors later in life (Pearce et al. 2012).

The protocol proposed hereby relies on the availability of patient 3D stereophotogrammetry scans, which is a non-invasive, non-ionising method to record 3D head shapes (Tenhagen et al. 2016); this method has become the standard for pre- and post-operative assessment of scaphocephaly correction and is slowly replacing the use of other imaging methods (CT, MRI) for the assessment.

The results are promising when comparing the anteroposterior and lateral dimensions of the CT and 3Dscan skull models, small differences were found (0.03 ± 3.12 mm for OFD and 2.03 ± 4.36 mm for BPD), proving that the outer surface of the calvarium can be reliably approximated by offsetting the head surface. The value of scalp thickness was similar to that found in Zapatero et al. (Zapatero et al. 2022)when measured at the euryon in patients affected by craniosynostosis. Domenech-Fernandez et al. (Domenech-Fernandez et al. 2021) found a skull thickness in control subjects aged 0-4 yo between 1.97 and 2.46 mm in the anterolateral, posterolateral and lateral calvarium, while a higher value in the anterior (up to 3.82 mm) and posterior skull (up to 4.78 mm). Gajawelli et al. (Gajawelli et al. 2020) performed neurocranial measurements in subjects up to 36 months and showed that in babies up to 1 year of age, thickness throughout the neurocranium is between 2 and 3 mm.

The simulations show a good agreement between the CT and S models in both localised expansion as well as extent of spring expansion. Both models achieve similar values of accuracy in capturing the spring kinematics, with the CT model and the S model replicating the on-table outcome within 3.9-5.5% of the full spring size (i.e. between 2.3 and 3.3 mm). In a previous study (Borghi et al. 2018), on a population of 18 patients, accuracy ranked between 0.5% to 3.9% (from immediate postop to second follow up) therefore the results are similar in this study’s cohort. Spring-induced increase in CI from PREOP was similarly captured by both models, with absolute difference between the two models equal to 1.2% ± 0.8% for POSTOP and 1.6% ± 0.6% at FU.

This study’s results have several implications. A novel methodology to create a simplified skull model using only head shape information was produced: the simplified models hereby created achieved sufficient accuracy from both the morphological and mechanical point of view, having dimensions similar to the skull as extracted from CTs and responding to spring force in a very similar way. This method relies on several heavy simplifications, such as assuming a constant thickness of both scalp and skull and a simplified geometry for coronal and lambdoid sutures. The assumption of constant skin thickness allowed the use of shell elements for the simulation of spring expansion, further cutting down simulation time. The assumption that sutures lie on a plane has been implicitly adopted in several other studies where a simplified skull model was created (Li et al. 2011; Li et al. 2013; Li et al. 2015); in this work, our group measured the angle between the nasion-tragions plane and the sutures to provide an accurate description of the suture location in comparison with the other anatomical structures and provide a protocol for replicating these anatomical structures without prior information on the subject’s calvarial anatomy.

While the CT model requires image processing to extract patient skull, sutures and fontanelles (which are manually segmented), resizing to account for growth (Borghi et al. 2020) and an average of 2.5h to run for a single surgical configuration (Borghi et al. 2020), the proposed method requires simple processing to produce the patient computer assisted design (CAD) model and the expansions simulation takes an average of 15 min. Furthermore, as the 3D stereophotogrammetry scans can be retrieved during preoperative consultations - a few days/hours before the surgery – it can be safely assumed that no significant growth has occurred between scan and surgery.

The main limitation of the current work is the fact that skull growth post-surgery is not taken into account. As stated elsewhere (Windh et al. 2008; Borghi et al. 2017), most of the spring expansion occurs within the first 1-2 days following the surgery. The current model is based on an optimised set of material properties (Borghi et al. 2020), which was optimized using spring expansion values up to 28 days post-insertion and therefore may not be reflective of the cranial elastic and viscoelastic properties at later stage (Coats and Margulies 2006). Other groups have in the past applied growth model to assess the outcome of other cranioplasty procedures (Libby et al. 2017; Marghoub et al. 2018) including springs (Cross et al. 2021). Future development of this model will include skull growth and validation could be performed using post-removal 3D stereophotogrammetry scans. In the present work, the two modelling modalities are compared both qualitatively (displacement pattern, Figure 5) and quantitatively (comparison of BPD and OFD at PREOP, POSTOP and FU time points). The predicted CI was also estimated and compared: although it has been criticised extensively as outcome measure (Fearon et al. 2017), it is still the most common index used for scaphocephaly diagnosis and correction assessment. Future work will address further validation using long-term postoperative 3D stereophotogrammetry using the methodology presented in our previous works (Borghi et al. 2018, 2020).

The present framework is designed for the prediction of sagittal craniosynostosis correction. Sagittal craniosynostosis is the most common presentation of non-syndromic craniosynostosis, accounting for up to 55% of all cases (Greenwood et al. 2014). Although springs are used for the treatment of other craniosynostosis presentation, such as unicoronal (Alford et al. 2018), lambdoid (Bozkurt et al. 2020) and syndromic craniosynostosis (Deliège et al. 2021), the present framework may require adaptation to be extended to be applied to preoperative modelling of other types of craniosynostosis correction.

We have here presented a protocol based on non-invasive imaging which can be used to simulate SAC surgery outcomes: this will help extend preoperative planning to a much larger patient population and will allow future model improvement as well as extension to the planning of other spring assisted surgeries, such as posterior vault expansion (Deliège et al. 2021) and lambdoid craniosynostosis correction (Bozkurt et al. 2020).

## References

[CIT0001] Abdel-Alim T, Iping R, Wolvius EB, Mathijssen IMJ, Dirven CMF, Niessen WJ, van Veelen M-LC, Roshchupkin GV. 2021. Three-dimensional stereophotogrammetry in the evaluation of craniosynostosis: current and potential use cases. J Craniofac Surg. 32(3):956–963. doi:10.1097/SCS.0000000000007379. 33405445

[CIT0003] Alford J, Derderian CA, Smartt JM. 2018. Surgical treatment of nonsyndromic unicoronal craniosynostosis. J Craniofac Surg. 29(5):1207. [accessed Nov 6]. doi:10.1097/SCS.0000000000004509. 29570518

[CIT0004] Borghi A, Rodriguez Florez N, Ruggiero F, James G, O’Hara J, Ong J, Jeelani O, Dunaway D, Schievano S. 2020. A population-specific material model for sagittal craniosynostosis to predict surgical shape outcomes. Biomech Model Mechanobiol. 19(4):1319–1329. doi:10.1007/s10237-019-01229-y. 31571084 PMC7424404

[CIT0005] Borghi A, Rodriguez-Florez N, Jeelani O, Dunaway D, Schievano S. 2018. Population-derived material properties for Craniosynostosis patients improve outcome predictions in Craniofacial Surgery. In: Proceedings of the 8th World Congress of Biomechanics.

[CIT0002] Borghi A, Rodriguez-Florez N, Rodgers W, James G, Hayward R, Dunaway D, Jeelani O, Schievano S. 2018. Spring assisted cranioplasty: a patient specific computational model. Med Eng Phys. 53:58–65. doi:10.1016/j.medengphy.2018.01.001. 29358032

[CIT0006] Borghi A, Schievano S, Florez NR, McNicholas R, Rodgers W, Ponniah A, James G, Hayward R, Dunaway D, Owase Jeelani NU. 2017. Assessment of spring cranioplasty biomechanics in sagittal craniosynostosis patients. J Neurosurg Pediatr. 20(5):400–409. doi:10.3171/2017.1.PEDS16475. 28841110

[CIT0007] Bozkurt S, Borghi A, Jeelani O, Dunaway D, Schievano S. 2020. Computational evaluation of potential correction methods for unicoronal craniosynostosis. J Craniofac Surg. 31(3):692–696. doi:10.1097/SCS.0000000000006186. 31977684

[CIT0008] Bozkurt S, Borghi A, van de Lande LS, Jeelani NUO, Dunaway DJ, Schievano S. 2020. Computational modelling of patient specific spring assisted lambdoid craniosynostosis correction. Sci Rep. 10(1):18693. doi:10.1038/s41598-020-75747-6. PMC7596227 33122820

[CIT0009] Bristol RE, Krieger MD, McComb JG. 2011. Normally shaped heads with no sutures, normally shaped heads with abnormal sutures, and abnormally shaped heads with normal sutures. J Craniofac Surg. 22(1):173–177. [accessed Jun 10]. doi:10.1097/SCS.0b013e3181f752c2. 21233761

[CIT0010] Coats B, Margulies S. 2006. Material properties of human infant skull and suture at high rates. J Neurotrauma. 23(8):1222–1232. [accessed Jan 28]. http://online.liebertpub.com/doi/abs/10.1089/neu.2006.23.1222. 16928180 10.1089/neu.2006.23.1222

[CIT0011] Cross C, Khonsari RH, Larysz D, Johnson D, Kölby L, Moazen M. 2021. Predicting and comparing three corrective techniques for sagittal craniosynostosis. Sci Rep. 11(1):21216. [accessed Jun 10]. doi:10.1038/s41598-021-00642-7. PMC8551239 34707183

[CIT0012] Deliège L, Misier KR, Bozkurt S, Breakey W, James G, Ong J, Dunaway D, Jeelani NUO, Schievano S, Borghi A. 2021. Validation of an in-silico modelling platform for outcome prediction in spring assisted posterior vault expansion. Clin Biomech (Bristol, Avon). 88:105424. [accessed Feb 15]. doi:10.1016/J.CLINBIOMECH.2021.105424. 34303069

[CIT0013] Didier RA, Kuang AA, Schwartz DL, Selden NR, Stevens DM, Bardo DME. 2010. Decreasing the effective radiation dose in pediatric craniofacial CT by changing head position. Pediatr Radiol. 40(12):1910–1917. doi:10.1007/s00247-010-1788-2. 20686761

[CIT0014] Domenech-Fernandez P, Yamane J, Domenech J, Barrios C, Soldado-Carrera F, Knorr J, Canavese F. 2021. Analysis of skull bone thickness during growth: an anatomical guide for safe pin placement in halo fixation. Eur Spine J. [30(2):410–415. [accessed Jun 10]. doi:10.1007/S00586-020-06367-X. 32248506

[CIT0015] Erasmie U, Ringertz H. 1976. Normal width of cranial sutures in the neonate and infant. Acta Radiol Diagn (Stockh). 17(5A):565–572. doi:10.1177/028418517601705A03. 983758

[CIT0016] Fearon JA. 2014. Evidence-based medicine: craniosynostosis. Plast Reconstr Surg. 133(5):1261–1275. doi:10.1097/PRS.0000000000000093. 24776557

[CIT0040] Fearon JA, Ditthakasem K, Herbert M, Kolar J. 2017. An Appraisal of the Cephalic Index in Sagittal Craniosynostosis, and the Unseen Third Dimension. Plast Reconstr Surg. 140(1):138–145. doi:10.1097/PRS.0000000000003422. 28654600

[CIT0017] Gajawelli N, Deoni S, Shi J, Linguraru MG, Porras AR, Nelson MD, Tamrazi B, Rajagopalan V, Wang Y, Lepore N. 2020. Neurocranium thickness mapping in early childhood. Sci Rep. 10(1):16651. [accessed Jun 10]. doi:10.1038/s41598-020-73589-w. PMC7538561 33024168

[CIT0018] Greenwood J, Flodman P, Osann K, Boyadjiev SA, Kimonis V. 2014. Familial incidence and associated symptoms in a population of individuals with nonsyndromic craniosynostosis. Genet Med. 16(4):302–310. doi:10.1038/gim.2013.134. 24071792 PMC4143991

[CIT0019] Jeelani NUO, Borghi A, Florez NR, Bozkurt S, Dunaway D, Schievano S. 2020. The science behind the springs: using biomechanics and finite element modeling to predict outcomes in spring-assisted sagittal synostosis surgery. J Craniofac Surg. 31(7):2074–2078. doi:10.1097/SCS.0000000000006865. 33003057

[CIT0041] Kapp-Simon KA, Speltz ML, Cunningham ML, Patel PK, Tomita T. 2007. Neurodevelopment of children with single suture craniosynostosis: A review. Childs Nerv Syst. 23(3):269–281. doi:10.1007/s00381-006-0251-z. 17186250

[CIT0042] Knight SJ, Anderson VA, Spencer-Smith MM, Da Costa AC. 2014. Neurodevelopmental outcomes in infants and children with single-suture craniosynostosis: a systematic review. Dev Neuropsychol. 39(3):159–186. doi:10.1080/87565641.2014.886690. 24742309

[CIT0020] Li Z, Hu J, Reed MP, Rupp JD, Hoff CN, Zhang J, Cheng B. 2011. Development, validation, and application of a parametric pediatric head finite element model for impact simulations. Ann Biomed Eng. 39(12):2984–2997. [accessed Jun 2]. doi:10.1007/s10439-011-0409-z. 21947736

[CIT0021] Li Z, Luo X, Zhang J. 2013. Development/global validation of a 6-month-old pediatric head finite element model and application in investigation of drop-induced infant head injury. Comput Methods Programs Biomed. 112(3):309–319. [accessed Nov 22]. doi:10.1016/j.cmpb.2013.05.008. 24008251

[CIT0022] Li Z, Park BK, Liu W, Zhang J, Reed MP, Rupp JD, Hoff CN, Hu J. 2015. A statistical skull geometry model for children 0-3 years old. PLoS One. 10(5):e0127322. [accessed Jun 10]. doi:10.1371/JOURNAL.PONE.0127322. 25992998 PMC4436309

[CIT0023] Libby J, Marghoub A, Johnson D, Khonsari RH, Fagan MJ, Moazen M. 2017. Modelling human skull growth: a validated computational model. J R Soc Interface. 14(130):20170202. [accessed Jul 24]. doi:10.1098/rsif.2017.0202. 28566514 PMC5454308

[CIT0024] Livingston MH, Igric A, Vogt K, Parry N, Merritt NH. 2014. Radiation from CT scans in paediatric trauma patients: indications, effective dose, and impact on surgical decisions. Injury. 45(1):164–169. doi:10.1016/j.injury.2013.06.009. 23845570

[CIT0025] Marghoub A, Libby J, Babbs C, Pauws E, Fagan MJ, Moazen M. 2018. Predicting calvarial growth in normal and craniosynostotic mice using a computational approach. J Anat. 232(3):440–448. [accessed Jul 24]. doi:10.1111/joa.12764. 29243252 PMC5807955

[CIT0026] Nowinski D, Di Rocco F, Renier D, SainteRose C, Leikola J, Arnaud E. 2012. Posterior cranial vault expansion in the treatment of craniosynostosis. Comparison of current techniques. Childs Nerv Syst. 28(9):1537–1544. doi:10.1007/s00381-012-1809-6. 22872270

[CIT0027] Obara H, Takahashi M, Kudou K, Mariya Y, Takai Y, Kashiwakura I. 2017. Estimation of effective doses in pediatric X-ray computed tomography examination. Exp Ther Med. 14(5):4515–4520. doi:10.3892/etm.2017.5102. 29104659 PMC5658718

[CIT0028] Pearce MS, Salotti J a, Little MP, McHugh K, Lee C, Kim KP, Howe NL, Ronckers CM, Rajaraman P, Sir Craft AW, et al. 2012. Radiation exposure from CT scans in childhood and subsequent risk of leukaemia and brain tumours: a retrospective cohort study. Lancet. 380(9840):499–505. [accessed May 25]. doi:10.1016/S0140-6736(12)60815-0. 22681860 PMC3418594

[CIT0029] Rodgers W, Glass GE, Schievano S, Borghi A, Rodriguez-Florez N, Tahim A, Angullia F, Breakey W, Knoops P, Tenhagen M, et al. 2017. Spring-assisted cranioplasty for the correction of nonsyndromic scaphocephaly: a quantitative analysis of 100 consecutive cases. Plast Reconstr Surg. 140(1):125–134. doi:10.1097/PRS.0000000000003465. 28338584

[CIT0030] Rodriguez-Florez N, Borghi A, Yauwan DD, Heuntinck P, Bruse JL, Tenhagen M, Göktekin ÖK, Angullia F, Schievano S, Dunaway DJ, et al. 2020. Three-dimensional calvarial growth in spring-assisted cranioplasty for correction of sagittal synostosis. J Craniofac Surg. 31(7):2084–2087. doi:10.1097/SCS.0000000000006863. 32804823

[CIT0031] Rodriguez-Florez N, Göktekin ÖK, Bruse JL, Borghi A, Angullia F, Knoops PGM, Tenhagen M, O'Hara JL, Koudstaal MJ, Schievano S, et al. 2017. Quantifying the effect of corrective surgery for trigonocephaly: a non-invasive, non-ionizing method using three-dimensional handheld scanning and statistical shape modelling. J Craniomaxillofac Surg. 45(3):387–394. doi:10.1016/j.jcms.2017.01.002. 28159480

[CIT0032] Sadrameli M, Mupparapu M. 2018. Oral and maxillofacial anatomy. Radiol Clin North Am. 56(1):13–29. [accessed Jun 10]. doi:10.1016/J.RCL.2017.08.002. 29157543

[CIT0033] Savoldi F, Tsoi JKH, Paganelli C, Matinlinna JP. 2018. The biomechanical properties of human craniofacial sutures and relevant variables in sutural distraction osteogenesis: a critical review. Tissue Eng Part B Rev. 24(1):25–36. doi:10.1089/ten.TEB.2017.0116. 28610544

[CIT0034] Sharma JD, O’Hara J, Borghi A, Rodriguez-Florez N, Breakey W, Ong J, Owase Jeelani N, Dunaway D, James G, Ãz F. 2018. Results following adoption of a modified melbourne technique of total scaphocephaly correction. [accessed Aug 7]. doi:10.1097/SCS.0000000000004593. 29771828

[CIT0035] Tenhagen M, Bruse JL, Rodriguez-Florez N, Angullia F, Borghi A, Koudstaal MJ, Schievano S, Jeelani O, Dunaway D. 2016. Three-dimensional handheld scanning to quantify head-shape changes in spring-assisted surgery for sagittal craniosynostosis. J Craniofac Surg. 27(8):2117–2123. doi:10.1097/SCS.0000000000003108. 28005766

[CIT0036] Wilkie AOM, Byren JC, Hurst JA, Jayamohan J, Johnson D, Knight SJL, Lester T, Richards PG, Twigg SRF, Wall SA. 2010. Prevalence and complications of single-gene and chromosomal disorders in craniosynostosis. Pediatrics. 126(2):e391–e400. doi:10.1542/peds.2009-3491. 20643727 PMC3535761

[CIT0037] Windh P, Davis C, Sanger C, Sahlin P, Lauritzen C. 2008. Spring-assisted cranioplasty vs pi-plasty for sagittal synostosis – a long term follow-up study. J Craniofac Surg. 19(1):59–64. doi:10.1097/scs.0b013e31815c94c8. 18216666

[CIT0038] Zakhary GM, Montes DM, Woerner JE, Notarianni C, Ghali GE. 2014. Surgical correction of craniosynostosis. A review of 100 cases. J Craniomaxillofac Surg. 42(8):1684–1691. [accessed May 17]. doi:10.1016/j.jcms.2014.05.014. 24969768

[CIT0039] Zapatero ZD, Morales CZ, Wes AM, Kalmar CL, Kosyk MS, Swanson JW, Bartlett SP, Taylor JA. 2022. A quantification of scalp thickness before and after posterior vault distraction osteogenesis. Plast Reconstr Surg. 149(2):462–466. [accessed Jun 10]. doi:10.1097/PRS.0000000000008767. 35077423

